# Recurrence and death after *Clostridium difficile* infection: gender-dependant influence of proton pump inhibitor therapy

**DOI:** 10.1186/s40064-016-2058-z

**Published:** 2016-04-11

**Authors:** Ophélie Dos Santos-Schaller, Sandrine Boisset, Arnaud Seigneurin, Olivier Epaulard

**Affiliations:** Infectious Disease Unit, Grenoble University Hospital, Grenoble, France; Faculty of Medicine, Grenoble Institute of Clinical, Biological and Epidemiological Infectiology, Grenoble, France; Laboratory of Bacteriology, Grenoble University Hospital, Grenoble, France; Quality Science and Medical Evaluation Unit, Grenoble University Hospital, Grenoble, France; Computational and Mathematical Biology, TIMC-IMAG UMR 5525, Grenoble, France; Service des Maladies Infectieuses, CHU de Grenoble, CS10217, 38043 Grenoble Cedex 09, France

**Keywords:** Proton pump inhibitor, *Clostridium difficile*, Death, Recurrence, Gender

## Abstract

**Goals:**

To determine whether patients with a pre-existing PPI treatment had a higher risk of poor evolution (recurrence or death) when diagnosed with a toxicogenic *Clostridium difficile* digestive infection.

**Background:**

Previous studies identified pump proton inhibitor (PPI) prescription as a risk factor for *C. difficile* infection. The influence of PPI on the outcome of *C. difficile* infection is controversial.

**Study:**

This was a retrospective monocentric cohort study. All cases of patients in our center with a symptomatic infection by a toxicogenic *C. difficile* strain during the years 2012 and 2013 were retrospectively analyzed. The primary endpoint was the occurrence of a recurrence or *C. difficile* infection -related death within 2 months after diagnosis.

**Results:**

373 patients were included in this study (198 men and 175 women), with a mean age of 70.1 ± 18.6 years (2–100 years). Fourteen (3.7 %) patients died secondarily to *C. difficile* infection (median survival time 5 days), and 88 (23.6 %) experienced recurrence (after a median delay of 30 days). One hundred and ninety eight (53.1 %) patients were already receiving PPI at the time of the *C. difficile* infection (including 156 patients with a prescription >1 month). When analyzing separately men and women, male patients were more likely to experience recurrence or death in case of pre-existing PPI prescription [HR = 2.32 (1.26–4.27)]; this was not observed in female patients [HR = 0.62 (0.31–1.22)].

**Conclusions:**

Pre-existing PPI therapy may increase the risk of recurrence or death in male patients with a toxicogenic *C. difficile* infection. PPI risk–benefit ratio should be carefully assessed.

## Background

*Clostridium difficile* infection (CDI) has become a common cause of acute diarrhea in adults. Over the last years, CDI incidence has increased three to eight times in the USA (Lessa et al. 2015; Gilca et al. 2010), along with the risk of complications (Pepin et al. 2004); in Europe, the rise of an hypervirulent *C. difficile* strain (027 or NAP1) has been observed, this strain being responsible for more severe clinical forms and more recurrences (Davies et al. 2014; Loo et al. 2005). CDI has various clinical forms, from relatively benign afebrile or febrile diarrhea, to simple colitis, pseudomembranous colitis, severe sepsis, toxic megacolon, and organ perforation; mortality rate of severe form reaches 50 % (Venugopal et al. 2013). Classical risk factors of CDI are recent hospitalization, antibiotic prescription, age over 65 years, and immunosuppression (Pacheco and Johnson 2013). In the last years, it has been suspected that proton pump inhibitor (PPI) therapy may be a risk factor for CDI (Kwok et al. 2012; Janarthanan et al. 2012). PPI are widely prescribed; in the USA, more than 11 million patients are treated with PPI (as a long term treatment) (Fashner and Gitu 2013), and overuse has been documented in Europe (Ramirez et al. 2010).

The purpose of this study was to determine whether patients with a pre-existing PPI treatment had a higher risk of CDI recurrence or CDI-related death when diagnosed with a toxicogenic *C. difficile* strain.

## Results

### Population

From January 2012 to December 2013, *Clostridium difficile* was detected in feces of 592 patients. Three hundred and seventy-three patients meeting the inclusion criteria (clinical symptoms including at least diarrhea, and fecal samples positive for toxicogenic *C. difficile*) were included. One hundred and ninety eight (53.1 %) patients were men and 175 (46.9 %) were women. The mean age was 70.1 ± 51.5–88.7 (2–100 years). Among the included patients, 5 were carrying the 027 *C. difficile* strain.

Among the 373 included patients, 198 (53.1 %) were receiving PPI before CDI; PPI therapy was initiated more than 1 month before CDI in 156 patients (41.8 %). Two hundred and seventy (72.4 %) patients received antibiotics within a month before the infection, 269 (72.1 %) had been hospitalized in the 3 months prior to the CDI, and 72 (19.3 %) were receiving long term immunosuppressive therapy (steroids, TNF-α blockers, anti-rejection therapy, cyclophosphamide, rituximab or azathioprine).

Concerning the CDI, 177 patients (47.4 %) had an afebrile diarrhea, 84 patients (22.5 %) had a febrile diarrhea, 70 (18.8 %) a colitis, and 42 (11.3 %) a severe colitis. A total of 16 % of patients were lost to follow-up. Fifty-three patients died in the year following the diagnosis of CDI, including 14 patients whose death was directly due to CDI, with a median survival time of 5 days (1–56 days). Recurrence of CDI occurred in 88 patients (23.6 %), with a median delay of 30 days (7–173 days). As most (80 %) of relapse occurred in the 2 months following CDI diagnosis in a recent study (McDonald et al. 2015), we chose to consider this period in our study. When considering only the 2 months following the initial CDI diagnosis, recurrence of CDI occurred in 74 patients (19.8 %), with a median delay of 27 days (7–55 days). The relative frequencies of CDI forms were not different in patients with and without pre-existing PPI therapy.

**Fig. 1:**
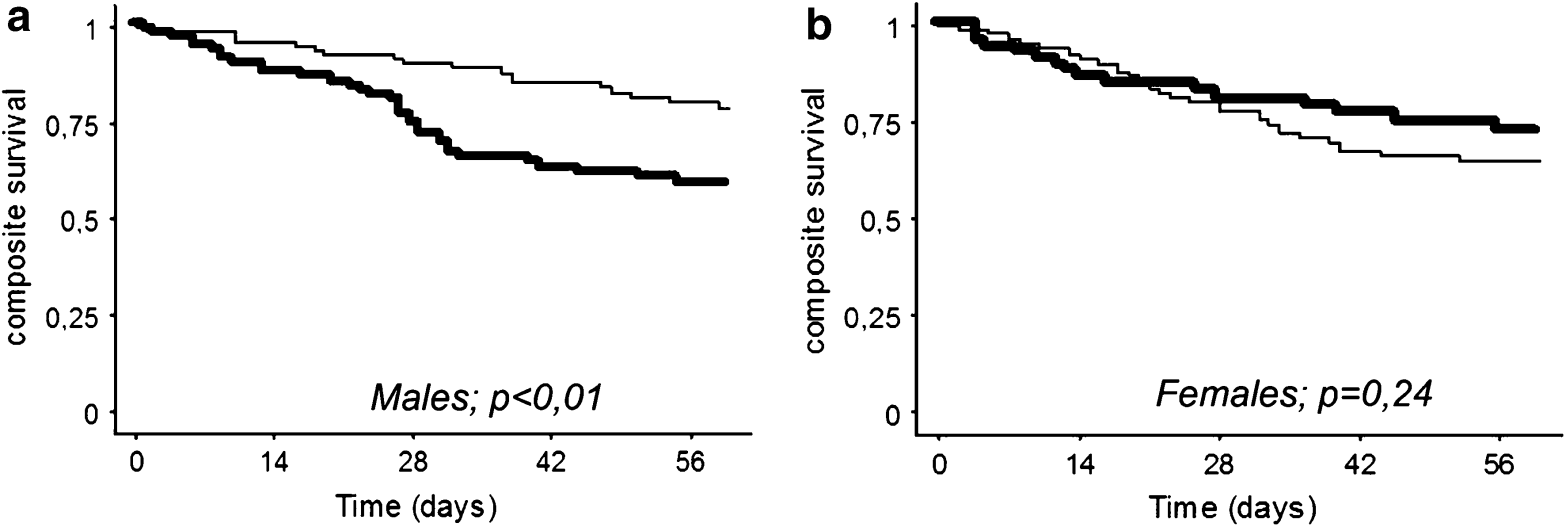
Kaplan–Meier curves of the composite risk of *C. difficile* infection (CDI) recurrence and CDI-related death in males (**a**) and females (**b**), in patients with proton pump inhibitor prescription during more than 1 month before CDI diagnosis (*bold line*) and other patients (*thin line*)

We then considered the composite risk of CDI recurrence or CDI-related death in the 2 months following the initial CDI diagnosis. In univariate analysis, this risk was higher in patients without immunosuppressive therapy (p = 0.02). Sex, age group (under/above 50 years), CDI clinical form and antibiotic therapy in the past month and hospital admission in the past 3 months were not associated with a distinctive evolution. Pre-existing PPI prescription during more than 1 month before CDI was not associated with a higher risk (p = 0.15); this difference was neither significant when considering all PPI prescription (p = 0.27). When considering separately males and females, this composite risk of recurrence or *C. difficile*-related death was significantly associated with pre-existing PPI prescription more than 1 month prior CDI in males (p < 0.01) but not in females (p = 0.24) (Fig. 1). This composite risk was also significantly associated with PPI prescription whatever the duration in males (p = 0.04) but not in females (p = 0.58). When considering the whole follow-up period, and not only 2 months, the difference in the composite risk was still significantly associated with PPI in men (p < 0.01) but not in women (p = 0.46); however, only few new events occurred after the first 2 months.

It is noteworthy that the rate of CDI-related death was too low to assess the association between this outcome alone and PPI prescription. When considering CDI relapse alone, the risk was still significantly associated with PPI in men (p < 0.01) but not in women (nonsignificant trend of lower risk of relapse, p = 0.16).

In multivariate Cox model including sex, PPI prescription >1 month, age group, previous hospitalization, previous antibiotic therapy, immunosuppressive therapy, and clinical form, PPI prescription was independently associated with a higher risk of recurrence of *C. difficile* related death in males [HR = 2.32 (1.26–4.27)] but not in females [HR = 0.62 (0.31–1.22)] (Table 1).

**Table 1:** Relative risk of CDI recurrence or CDI-related death: multivariate analysis

	RR of CDI recurrence or CDI-related death	95 % confidence interval
PPI prescription^a^—women		
No	1.00	
Yes	0.62	0.33–1.22
PPI prescription^a^—men		
No	1.00	
Yes	2.32	1.26–4.27
Sex		
Female	1.00	
Male	0.51	0.28–0.94
Age		
<50 years	1.00	
>50 years	1.53	0.68–3.44
Previous hospitalization		
No	1.00	
Yes	1.71	0.98–2.97
Previous antibiotic therapy		
No	1.00	
Yes	0.95	0.59–1.52
Immunosupressive therapy		
No	1.00	
Yes	0.32	0.15–0.68
Clinical form		
Diarrhea	1.00	
Colitis	1.10	0.68–1.78

^a^Pre-existing PPI therapy more than 1 month before

## Discussion

Proton pump inhibitors are widely prescribed; however, their benefits may not deserve such common use (Katz 2010). They may be frequently useless, and may be associated with poorly appraised adverse effects, including osteoporosis (Gray et al. 2010) and infections. Indeed, prospective and retrospective studies suggested that PPI may increase the risk of both community-acquired pneumonia and intensive care unit-acquired pneumonia (Eom et al. 2011), although this was not always observed (Filion et al. 2014). A meta-analysis also suggested that PPI prescription enhances the risk of spontaneous bacterial peritonitis in patients with ascites (Trikudanathan et al. 2011). *Clostridium difficile* infections may also be more frequent in patients receiving PPI; a large prospective study (Loo et al. 2011) observed that PPI prescription was associated with a relative risk of CDI and colonization of respectively 2.64 and 1.71; and a study of critically ill patients (Buendgens et al. 2014) showed that PPI prescription was associated with a relative risk of 3.11 of developing CDI in the intensive care unit. Meanwhile, two meta-analyses (Tleyjeh et al. 2012; Kwok et al. 2012) and a case–control study (Leonard et al. 2012) do not support this association.

Some previous studies already explored the association between PPI prescription and CDI outcome. In a monocentric USA study (Freedberg et al. 2013), PPI were associated with higher 90-day mortality in patients with CDI, but not with recurrence. Other studies observed that PPI prescription was associated with CDI recurrence in a USA population (hazard ratio 1.4) (Linsky et al. 2010), in a Canadian population (hazard ratio 1.5) (McDonald et al. 2015), and in a Korean population (Kim et al. 2012).

Our study also suggests that PPI prescription enhances the risk of CDI recurrence, but only in males. Gender has been associated with different CDI prognosis: in two USA studies, CDI were slightly more frequent in females (Lessa et al. 2015), and *C. difficile*-associated death was nearly twice higher in female (Smith et al. 2015), but no difference was observed in another USA study (Boone et al. 2012). Conversely, a French study observed a higher frequency of severe forms in males (Khanafer et al. 2013). Our results also suggest that there is an independent link between male gender and outcome.

The mechanisms leading to more frequent and/or more recurrent CDI are only suspected. Stomach low pH probably acts as a chemical defense against ingested microorganism, and drugs interfering with proton secretion are likely to limit this protection. Interestingly, a recent study (Freedberg et al. 2015) observed that during PPI use, patient gut microbiome was modified, with a higher proportion of bacterial taxa associated with CDI, suggesting that PPI may not only act on the ingested pathogens, but also on the normal gut flora.

Our study has several limitations, being monocentric and retrospective. However, as all the patients were diagnosed in a single center, it ensured that CDI diagnosis was homogenous among the whole population. To prevent some of the bias induced by the retrospective collection of data, we chose to only consider the events occurring within 2 months after the CDI diagnosis, as more delayed events may be missed in a retrospective study. It should also be noted that the infectious disease team of our institution is routinely advised in case of toxigenic *C. difficile* detection in the other medical unit of the hospital, meaning that it is unlikely that diagnosed CDI recurrence have been missed.

## Conclusion

Our study suggests that PPI use is associated with a higher risk of CDI recurrence and CDI-associated death in males. PPI prescription must be carefully appraised due to such adverse effects. Moreover, PPI should be withdrawn in patients with a diagnosis of CDI, at least temporarily.

## Methods

### Study design

The study was based on a retrospective cohort of patients diagnosed with a symptomatic infection by a toxicogenic *C. difficile*.

### Ethical consideration

This study was specifically approved by the regional ethical authority (Comité de Protection des Personnes Sud-Est V).

### Patient inclusion

We retrospectively included patients with diarrhea and fecal isolation of a toxicogenic *Clostridium difficile* in Grenoble University Hospital between 1st January 2012 and 31 December 2013. Patients without digestive symptoms or with a non-toxicogenic *C. difficile* strain were not included. Children aged less than 24 months were excluded, as the high rate of asymptomatic colonization in infants may interfere with diagnosis accuracy.

### *C*. *difficile* infection diagnosis

Liquid stool samples were submitted for detection of toxigenic *C. difficile* by a two-step algorithm. The *C. difficile* algorithm began with the C. DIFF QUICK CHEK^®^ test (TechLab), a membrane-bound enzyme immunoassays (EIA) that detects the glutamate dehydrogenase (GDH) antigen of *C. difficile*. From January to Mars 2012, a positive GDH result triggered the second-step TOX A/B QUICK CHEK^®^ (TechLab) EIA for toxins A and B detection. For specimens with negative EIA results (GDH positive, Toxins A/B negative), a culture was performed and the TOX A/B QUICK CHEK^®^ test was renewed from colonies. Subsequently, from April 2012, the second step of algorithm consisted of a direct stool PCR to detect the tcdB gene (Xpert^®^*C. difficile*, Cepheid).

### Data collection

Patient characteristics were retrospectively collected in medical files: age, sex, immunosuppressive therapy, hospitalization within 3 months prior to CDI diagnosis, antibiotic treatment within 1 month prior to CDI diagnosis, and PPI therapy prior to CDI diagnosis as well as whether this treatment was established more than 1 month before CDI. *C. difficile* antibiotic therapy was also collected and four clinical forms were considered: afebrile diarrhea, febrile diarrhea, colitis, and severe colitis. Complications, CDI recurrence, and/or *C. difficile*-related death were collected during the 2-months following CDI diagnosis.

The primary endpoint was the occurrence of a CDI recurrence or a CDI-related death within 2-months after diagnosis.

### Data analysis

Survival and recurrence-free survival were analyzed by the Kaplan–Meier method and Log-rank tests. The effect of PPI on the primary endpoint was adjusted for confounding factors using a Cox proportional hazard model. Potential confounding factors included were as follows: sex, age group (<50; ≥50 years), previous hospitalization, previous antibiotic therapy, immunosuppressive therapy, and clinical form. We tested for significance an interaction term between sex and PPI to assess if the effect of PPI was different in men and women.

Two-sided p values less than 0.05 were considered statistically significant. Analyses were performed using Stata version 13.0 (Stata Corporation, College Station, TX, USA).

## Abbreviations

CDI: *Clostridium difficile* infection
PPI: Proton pump inhibitor
